# Quadratus Lumborum Block As A Single Anesthetic Method For Laparoscopic Totally Extraperitoneal (Tep) Inguinal Hernia Repair: A Randomized Clinical Trial

**DOI:** 10.1038/s41598-020-65604-x

**Published:** 2020-05-22

**Authors:** Murillo de Lima Favaro, Silvio Gabor, Diogo Barros Florenzano Souza, Anderson Alcoforado Araújo, Ana Luiza Castro Milani, Marcelo Augusto Fontenelle Ribeiro Junior

**Affiliations:** 1 Professor of General Surgery and Surgical Technique at the University Santo Amaro, São Paulo, Brazil; 2 Professor of Anesthesiology at the University Santo Amaro, São Paulo, Brazil; 3 Fellow of Anesthesiology at the University Santo Amaro, São Paulo, Brazil; 4 Full Professor of General Surgery at the University Santo Amaro, São Paulo, Brazil

**Keywords:** Surgery, Randomized controlled trials

## Abstract

Minimally invasive surgery for inguinal hernia repair is advantageous in terms of return to usual activities and lower rates of chronic pain; however, it requires general anesthesia. This study sought to analyze the benefits of ultrasound guided locoregional anesthesia of the quadratus lumborum muscle (QL block) as a single anesthetic technique for endoscopic totally extraperitoneal (TEP) inguinal hernia repair with regard to postoperative pain, length of hospital stay, and hospital cost. A total of 46 patients, aged 18 to 80 years, with unilateral inguinal hernia, one group that received general anesthesia and one that received sedation and QL block for TEP inguinal hernia repair. In the 46 patients the median pain score 6 hours after surgery was significantly lower (2 versus 4) among the QL block group than among the group receiving general anesthesia. Consequently, the former group showed a briefer median hospital stay (6 versus 24 hours, respectively). The anesthesia and hospital costs were also lower for the QL block group, with median reductions of 64.15% and 25%, respectively. QL block is a safe and effective option for patients undergoing TEP inguinal hernia repair, given the observed reduction in early postoperative pain, briefer hospital stay, and decreased anesthesia and hospital costs.

## Introduction

More than 20 million patients undergo inguinal hernia repair each year worldwide, making it one of the most frequently performed surgeries^1,2^.

Inguinal hernia treatment has several technical approaches. Open surgery remains widely performed, although recent studies have shown that laparoscopic repair is a useful option because it has the following advantages: lower postoperative pain, lower analgesic intake, early return to physical and occupational activities, and lower rates of long-term complications such as chronic pain. The rate of recurrence is similar, regardless of the access route^1–7^.

The advantage of endoscopic totally extraperitoneal (TEP) inguinal hernia repair as described by Dulucq in 1992^8^ is that it does not require abdominal cavity entry. Therefore, the duration of this surgery is briefer, and the risk of unnoticed injury to intrabdominal structures is reduced^9,10^.

As a disadvantage, minimally invasive surgery for inguinal hernia repair requires general anesthesia, which increases the risks for patients with comorbidities as well as raises costs and lengthens hospital stays. Thus, this technique runs counter to the current trend to prioritize day hospital procedures to reduce costs in both public and private healthcare^11,12^.

Ultrasound (US)-guided locoregional anesthesia is highly beneficial for multimodal analgesia after abdominal surgery and for complete anesthesia in certain procedures. Several transversus abdominis plane (TAP block) techniques, including the most common US-guided approach in which the probe is placed on the midaxillary line, provide analgesia primarily to dermatomes T10–L2. However, the spread of the anesthetic agent can be irregular on occasion, resulting in variable analgesia across the surgical wound^13–15^.

A relatively new US-guided approach involving the quadratus lumborum (QL) muscle might ensure the wider spread of the local anesthetic agent (T5–L1) compared with a TAP block and therefore lead to better analgesia^13^.

The QL block was first described by Rafael Blanco at the 2007 annual meeting of the European Society of Regional Anesthesia (ESRA)^16^. The basic concept of the QL block is the deposition of a local anesthetic solution adjacent to the anterolateral aspect of the QL muscle. The spread pattern obtained is similar to that of the landmark-based TAP block, in that there is subsequent extension into the thoracic paravertebral space. Borglum *et al*. originally described placing the needle tip anterior to the QL muscle^17,18^ using their transmuscular approach. This technique was later refined by applying a posterior approach, named the “shamrock method” (with the erector spinae, QL, and psoas muscles as the leaves and the L4 transverse process as the stem). Administering local anesthesia between the QL and psoas muscles ensures a reliable spread into the thoracic paravertebral space^19^. This interfascial plane is closely related to the lumbar paravertebral space and the multiple nerves in their most central location, especially in our study, to the nerves of the inguinal canal: genitofemoral, iliohipogastric, ilioinguinal and lateral femoral cutaneous.

The QL block has four approaches, each with its own nomenclature. These approaches are the posterior, anterior, lateral, and transmuscular^19^.

Given the potential advantages of a QL block, the present study evaluated the benefits of QL block anesthesia alone with regard to postoperative pain, length of hospital stay, and hospital costs among patients undergoing laparoscopic TEP inguinal hernia repair.

## Methods

The sample was composed of 46 patients with unilateral inguinal hernia, American Society of Anesthesiologists (ASA) physical status I–II, aged 18 to 80 years old, without signs indicative of severity. A quality of life (QoL) questionnaire – EuraHS-QoL was administered during outpatient visits before surgery, the results of which revealed patterns of pain and restricted activities. All the patients were referred from the ambulatory of the group of abdominal hernias of the general hospital of the Grajaú and were operated in this same hospital and an informed consent was obtained from all patients^20^.

The study was performed according to the Ethics Committee of the Sirio Libanes Social Responsibility Institute: **CAAE (Presentation Certificate for Ethical Appreciation):** 90634818.5.0000.5447 and approval of *Plataforma Brasil* - a unified national database of human research records for the entire *CEP/Conep* (National Committee of Ethics and Research) system – **Approval Number:** 2.798.754 and the Committee of Randomized Trials – *ReBEC* (Brazilian Registry of Clinical Trials): **Trial Registration number** - RBR-4xtw6z, date of registration - 03/15/2019 and URL - http://www.ensaiosclinicos.gov.br/rg/RBR-4xtw6z/.

**Figure 1:**
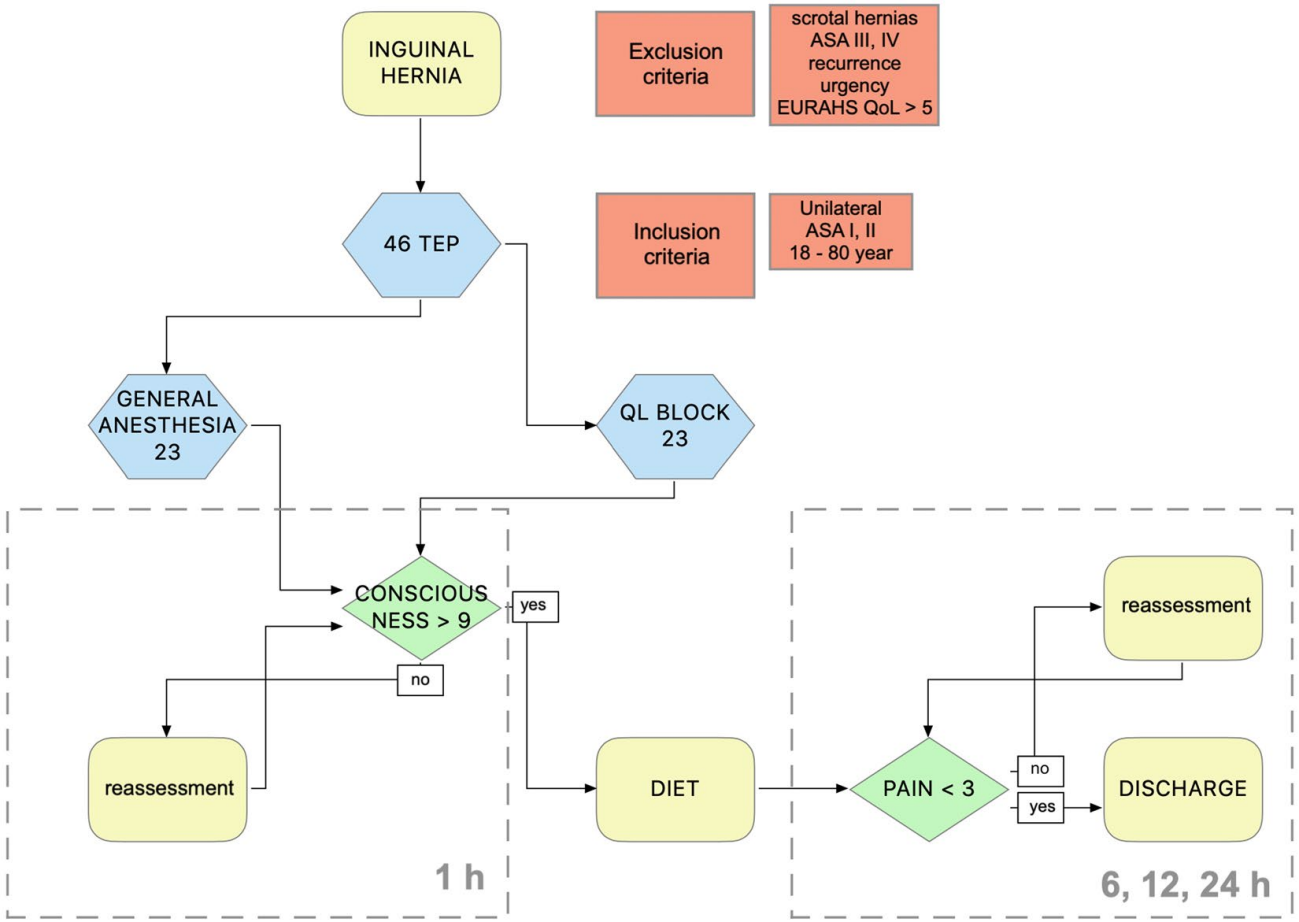
Flowchart representing patient selection and randomization.

Before surgery, the patients were randomly assigned to two groups using the program Randomizer for Clinical Trials™: One received general anesthesia, and the second received sedation and QL block. All patients underwent laparoscopic TEP inguinal hernia repair. The exclusion criteria were the patient’s age below 18 years and above 80, scrotal hernias, ASA physical status greater than 3, patients with preoperative pain at rest greater than 5 due to the quality of life classification (EuraHS Quality of life score), recurrent hernias or urgency. (Fig. 1).

The following standardized anesthetic procedure was applied.

All patients were duly monitored before surgery via cardioscopy, pulse oximetry, noninvasive blood pressure measurement (every 5 minutes), and the bispectral index (BIS; a brain monitoring system) to assess sedation level. Sedation was achieved using a continuous infusion of fentanyl citrate (50 mcg) and 1% propofol.

For the locoregional anesthesia group, antisepsis included cleansing of the skin of the lumbar area and iliac fossa using chlorhexidine alcohol. A high-frequency linear transducer (10 MHz) was used to guide the anesthetic block puncture, which was placed on Petit’s triangle; identification of the QL muscle was confirmed on US. A 22 G, 90-mm-long BD™ Whitacre spinal needle was placed in-plane on the anterolateral margin of the QL muscle at its junction with the transversalis fascia. A rectus sheath block was performed with the same transducer and needle. The needle was placed in-plane on the lateral margin of the rectus abdominis muscle before the emergence of the anterior branch of the intercostal nerve. The local anesthetic agent selected was 0.5% ropivacaine hydrochloride given its unique pharmacological properties, especially its lower cardiotoxic profile. Volumes of 30 ml and 20 ml were administered to the QL muscle and the rectus sheath block, respectively^21^.

Before starting the surgical procedure, an assessment of the quality of the block was performed routinely with the performance of a painful stimulus in the area that would be operated on, even with the patient under mild sedation. The procedure continued if the patient did not report pain when asked. After that, sedation was gradually increased, being monitored with the BIS, hemodynamic changes and eventual patient complaints.

Complications of QL block are local hematoma, intoxication by the local anesthetic (which can be avoided by not exceeding the dose by the patient’s limit weight) and the ineffectiveness of the block. Blockade failure is resolved by converting to general anesthesia using a higher dose of intravenous opioids, neuromuscular blockers, using a laryngeal mask as the first option and with orotracheal intubation as the second option.

In the general anesthesia group, induction was performed with 3 mcg/kg fentanyl, 3 mg/kg propofol, and 0.5 mg/kg atracurium, followed by orotracheal intubation. Maintenance was achieved with inhaled sevoflurane under BIS monitoring. At the end of the surgery, 1 mg atropine and 2 mg neostigmine were administered for neuromuscular block reversal.

The duration of anesthesia until the start of the surgical procedure was recorded, as were the medications used in each group and the presence of any complications.

As a part of the protocol, the patients received antibiotic prophylaxis with 2 g cephazolin during induction.

Intraoperative period: TEP was performed, the initial space was established via dissection under visualization with the scope. Following identification of anatomical landmarks, the hernia was reduced, and the preperitoneal space was dissected until a polypropylene mesh (15 × 13 cm) could be introduced to occlude Fruchaud’s myopectineal orifice. The mesh was not fixated. Next, after hemostasis revision, the pneumoperitoneum was emptied while the scope was kept inside the preperitoneal space for visualization of the peritoneum. The mesh was pushed against the anterior wall to ensure that it was correctly positioned^8,22,23^.

The same surgical technique was used for all procedures. No patient was excluded from the study for technical reasons or complications.

After the end of the surgery, the patients were transferred to the recovery room, where their levels of consciousness (Aldrete and Kroulik score)^24^ and pain (visual analog scale; VAS) were assessed^25^. The patients were monitored every 10 minutes until their consciousness scores reached 9 or higher; the time when this level was reached was recorded, pain was then assessed, and the patients were offered a light snack.

The patients received dipyrone 2 g intravenously (IV) during anesthesia and 6 hours later as per the protocol. Ketoprofen (100 mg) was administered IV during the surgery, and morphine (2 mg) was administered IV when the pain score was over 6. Patients also received 1 g dipyrone orally every 6 hours starting 6 hours after the surgical procedure (regardless of release or hospital stay) for 2 days and after that only if they were in pain. The use of anti-inflammatory drugs was avoided. It was not necessary to use additional opioids and antiemetics in any of the groups.

The patients were assessed for discharge 6 hours after surgery at the surgical ward by a surgeon who was blind to the study protocols. The patients were discharged when their pain scores were less than 3.

Patients not cleared for discharge 6 hours after surgery were assessed again within 12 to 24 hours after the procedure. Patients discharged early (prior to the previously described period) were contacted by telephone 24 hours after surgery to determine their level of pain^26^.

### Statistical analysis

Continuous and ordinal variables were compared using a Gaussian curve. The Kolmogorov-Smirnov (KS) distance and the Shapiro-Wilks tests indicated that the data were nonnormal; therefore, they were expressed as medians and percentiles (25th–75th). The Mann-Whitney U test with a Bonferroni correction was used to compare the two independent groups. Categorical data were expressed as absolute (n) and relative (%) frequencies. Contingency matrices were analyzed using Pearson’s chi-square test; complex matrices (2 × 3, 3 × 4, and so on) were transformed into simple matrices (2 × 2) to achieve a more accurate determination of causality. The alpha level (i.e., the probability of committing a type I error) was set to 5%, and the beta level (i.e., the probability of committing a type II error) was set to 20%.

## Results

The sample included 46 patients who were randomly allocated into two groups with 23 patients each. No patient had to be excluded based on the duration of surgery or complications during surgery. The median age of the patients was 45 years, and their median body mass index (BMI) was 23.9 kg/m^2^. The patients’ characteristics were similar between the two groups (Table 1) except for smoking, i.e., all five smokers received general anesthesia.

**Table 1:** Patient characteristics.

Characteristics	General anesthesia	QL	p
Number	23	23	
Age, median ± SD	45 ± 14.79	36 ± 18.25	0.150
BMI (kg/m^2^), median ± SD	24.2 ± 3.6	23.5 ± 3.35	0.361
SAH n (%)	5 (21.7%)	4 (17.4%)	0.710
DM, n (%)	1 (4.3%)	3 (13%)	0.295
Smoker, n (%)	5 (21.7%)	0	0.018*
Antiplatelet agents, n (%)	1 (4.3%)	1 (4.3%)	1
CHF n (%)	0	1 (4.3%)	0.312
Scar in the lower abdomen, n (%)	1 (4.3%)	4 (17.4%)	0.155

QL: quadratus lumborum anesthesia, SD: standard deviation, SAH: systemic arterial hypertension, DM: diabetes mellitus, CHF: congestive heart failure; *statistically significant

None of the analyzed surgical aspects, including type of hernia, defect size, laterality, type of instruments, and need for mesh fixation, differed significantly between the groups, indicating that the groups were homogenous (Table 2). Most of the patients (65.22%) who received general anesthesia had a direct hernia less than 3 cm in diameter. In turn, 43.48% of the patients in the QL block group had an indirect hernia measuring less than 3 cm in diameter. The median defect size was the same for both groups (4 cm^2^), and the side distribution was also similar.

**Table 2:** Surgical characteristics.

Characteristics	General anesthesia	QL	p
Number	23	23	
**EHS**			
L1 (lateral < 1.5 cm)	2 (8.7%)	4 (17.39%)	0.188
L2 (lateral 1.5–3 cm)	4 (17.39%)	10 (43.48%)	
L3 (lateral > 3 cm)	0	0	
M1 (medial < 1.5 cm)	1 (4.35%)	1 (4.35%)	
M2 (medial 1.5–3 cm)	15 (65.22%)	7 (30.43%)	
M3 (medial > 3 cm)	1 (4.35%)	1 (4.35%)	
**SIDE**			
Right	13 (56.5%)	10 (43.5%)	0.376
Left	10 (43.5%)	13 (56.5%)	
Instruments 5 mm	16 (69.6%)	12 (52.2%)	0.227
Instruments 3 mm	7 (30.4%)	11 (47.8%)	
Mesh fixation no	22 (95.7%)	21 (91.3%)	0.550
Mesh fixation yes	1 (4.3%)	2 (8.7%)	
Size (cm^2^) median ± SD	4 ± 3.45	4 ± 3.28	0.246

QL: quadratus lumborum anesthesia, EHS: inguinal hernia classification according to the European Hernia Society, L: lateral (indirect), M: medial (direct), SD: standard deviation.

The results of the quality of life questionnaire administered before surgery revealed similar patterns of pain and activity restriction between the groups (Table 3). The median score for pain during outdoor activities and sports did not significantly differ between groups (5 and 6, respectively). All other median scores were lower for the QL block group; however, these differences were not significant for the variables pain at rest, during daily activities, and restriction on daily activities.

**Table 3:** Comparison of the quality of life score by type of anesthesia.

Anesthesia	General			QL			Mann-Whitney
	Median	25%	75%	Median	25%	75%	p
At rest*	2.0	0.0	5.0	0.0	0.0	4.0	0.343
During activities*	5.0	2.0	7.0	4.0	3.0	7.0	0.603
Worst pain in the last week*	5.0	2.0	9.0	4.0	0.0	6.0	0.267
Activities of daily living*	2.0	0.0	5.0	0.0	0.0	4.0	0.916
Outdoor activities*	5.0	0.0	7.0	5.0	0.0	7.0	0.839
During sports *	6.0	0.0	10.0	6.0	3.0	10.0	0.615
During heavy activities*	7.0	0.0	10.0	7.0	3.0	9.0	0.621

QL: quadratus lumborum anestesia, * quality of life (QoL) questionnaire – EuraHS-QoL.

The median duration of anesthesia was similar between the groups. All surgeries followed the same standardized protocol. The mean duration of surgery was 60 minutes for the group that received general anesthesia and 50 minutes for the QL block group; this difference was not significant. The median postanesthesia recovery time was 20 minutes for the group that received general anesthesia and 10 minutes for the QL block group; this difference was significant (Table 4).

**Table 4:** Comparison of the intra and postoperative time variables by type of anesthesia.

Anesthesia	General			QL			Mann-Whitney
	Median	25%	75%	Median	25%	75%	p
Anesthesia duration (min)	20	19	34	20	19	34	**0.614**
Surgery duration (min)	59	45	60	50	29	70	**0.790**
Time to POR	20	15	25	10	10	20	0.001*
Length of stay (h)	24:00	12:00	24:00	6:00	6:00	6:00	<0.001*

QL: quadratus lumborum anesthesia, min: minutes, POR: postoperative recovery, h: hours,

*Significant.

Pain 6 hours after surgery was significantly lower (2 versus 4) for the QL block group, resulting in a briefer median hospital stay of 6 hours for the QL group compared with 24 hours for the group that received general anesthesia (Table 5).

**Table 5:** Comparison of pain according to the VAS by type of anesthesia.

Anesthesia	General			QL			Mann-Whitney
	Median	25%	75%	Median	25%	75%	P
Pain 1 h	2	0	4	2	0	3	0.241
Pain 6 h	4	3	4	2	0	2	<0.001*
Most pain	4	3	4	2	0	2	<0.001*
Least pain	2	1	2	2	0	2	0.645

VAS: visual analog scale, QL: quadratus lumborum anesthesia; *Significant.

The use of dipyrone in the postoperative period after hospital discharge was similar in both groups and no additional medication was used in both groups.

The median anesthesia and hospital costs were 64.15% and 25% lower, respectively, for the QL block group than the general anesthesia group (Table 6).

**Table 6:** Analysis of direct costs by type of anesthesia.

Anesthesia	General			QL			Mann-Whitney
	Median	25%	75%	Median	25%	75%	p
Anesthesia cost (US$)	140.87	96.26	200.93	90.38	79.67	93.92	<0.001*
Hospital cost (US$) *	400.00	300.00	400.00	100.00	100.00	100.00	<0.001*
Anesthesia + hospital cost (US$) **	496.26	440.04	540.87	190.38	181.45	194.52	<0.001*

QL: quadratus lumborum anesthesia. Data are expressed as medians and quartiles and analyzed using the Mann-Whitney test.

*Significant.

**The mesh cost was not included; all patients received the same type of mesh.

In addition to evaluations during the 24-hour post-surgical period, daily evaluations were carried out after these 24 hours until the first return on the 7th postoperative day. These evaluations were made via telephone and in person on the return of the 7th postoperative day. No difference was found between groups in this postoperative evaluation even when different periods were selected (1–3 days, 1–5 days, 3–7 days). A validated questionnaire was used to show the homogeneity of the groups in the preoperative evaluation and this same questionnaire was repeated in the 7-day, 30-day, 90-day and annual return, with no significantly difference being found between the groups in this evaluation.

## Discussion

The search for cost-effective options to treat inguinal hernia is ongoing because this surgery is one of the most often performed worldwide. The current consensus for the treatment of inguinal hernia developed by the union of the world’s largest hernia societies, indicates that laparoscopic repair shows better outcomes in terms of return to usual activities and a lower frequency of chronic pain compared with open surgery. However, the latter technique still has the advantage with regard to cost and length of hospital stay. One of the reasons for these differences in cost and length of hospital stay is that laparoscopic repair is performed under general anesthesia^1–6,27–30^.

Dhankhar *et al*. compared TEP under general anesthesia with open surgery under local anesthesia and found advantages for the latter, including a briefer surgery duration, smaller mesh size, and lower cost of anesthesia. In other words, open surgery is advantageous for unilateral noncomplicated inguinal hernia, especially in countries with scarce resources. These findings notwithstanding, the rates of hematoma, seroma, and delayed wound healing were higher in the group receiving open surgery. In our study, the median duration of surgery was briefer than that of the open surgery reported by Dhankhar *et al*., who found that pain, analgesic intake, and C-reactive protein (CRP) levels were lower among those who underwent laparoscopic repair. Considering the current findings of briefer surgery duration and lower anesthesia cost in combination with all of the advantages of laparoscopic surgery, we believe that we have contributed to improving cost-effectiveness^29^.

As is known, avoiding general anesthesia, or complementing it with a local anesthetic block, can reduce or eliminate the indication of opioids, with a consequent reduction of the complications associated with these drugs (e.g., postoperative nausea, paralytic ileus, sleepiness, and others). In their randomized study, Arora *et al*. reported a reduced need for opioids and analgesics as well as less pain both at rest and upon movement among patients receiving laparoscopic hernia repair with a TAP block^14,31–34^.

Avoiding general anesthesia also reduced the rates of problems related to mechanical ventilation (e.g., alveolar microinjury) and those exhibited by patients with chronic obstructive pulmonary disease (COPD)^5,35–37^. Hausman *et al*. conducted a retrospective study that analyzed more than 5,000 patients with COPD receiving several surgeries, including hernia repair, divided into two groups according to the type of anesthesia received. The results showed a higher risk of pneumonia and prolonged orotracheal intubation in the group receiving general anesthesia compared with the group receiving spinal anesthesia. In addition, the benefits of locoregional anesthesia via a TAP block or a QL block were the same as those of spinal anesthesia (e.g., better analgesic effect and no need of ventilatory support)^36^.

The choice of QL block, which does not require orotracheal intubation, as a single anesthetic procedure might enable laparoscopy for patients with contraindications to general anesthesia. Although spinal anesthesia also affords this advantage, it is associated with undesirable effects such urinary retention, delayed ambulation, and a briefer analgesic effect compared with QL block, which might delay hospital discharge and increase the total cost of the procedure^5,38^. In a study of 94 patients receiving laparoscopic hernia repair under spinal anesthesia, Tzovaras *et al*. found that while this procedure was safe, it was accompanied by 33%, 16%, and 13% rates of urinary retention, bradycardia, and hypotension, respectively. Although some studies have considered urinary retention as a relevant postoperative factor^36,38^, no patient exhibited this condition in the present study.

Random allocation resulted in homogeneous groups; however, bias was observed with regard to one aspect (smoking), which was significantly more frequent in the group receiving general anesthesia.

No between-group difference was observed with regard to the duration of anesthesia. This finding indicates that the duration of a QL block, when performed by a duly trained team, is similar to that of general anesthesia and therefore does not increase the total duration of the procedure. Our results in this regard disagree from those of most studies, such as those by Arora *et al*., Kim *et al*., and Meyer *et al*., all of whom found that the duration of the procedure was longer. The reason is that, in the present study, QL block was used as anesthetic procedure and not as an adjuvant. In the aforementioned studies, the duration of QL block was added to that of the procedure performed on the control group^34,39,40^.

Meyer *et al*. mentions in 2015 that the lack of muscle relaxation due to the non-use of neuromuscular blocking agents during general anesthesia did not interfere negatively in inguinal hernioplathy using the TEP technique. A loss of visualization of the surgical field could be expected due to the difficulty in making the pneumopreperitoneo and consequently an increase in the surgical time, without the muscle relaxation generated by neuromuscular blockers. But these consequences were not found in most studies that did not use neuromuscular blockers. This is evident in our study in which the average duration of surgery was 10 minutes shorter in the QL block group^39^.

Peritoneal microperforations are common during TEP, which enable the passage of carbon dioxide (CO_2_) from the preperitoneal space into the abdominal cavity. This occurrence is one of the major hazards associated with locoregional anesthesia. In the present study, pneumoperitoneum occurred toward the end of surgery in patients of both groups, which indicates the occurrence of small peritoneal lesions. However, this development did not interfere with the surgical procedure or postoperative recovery nor did it required increased sedation or amount of anesthetic agent^9,41^. Muzio *et al*. suggested that peritoneal lesions increase the rate of surgery conversion and duration but not the level of pain or rate of late complications. No patient in our study required conversion to open surgery^41^.

We found a significant difference in the assessments performed starting 6 hours after surgery. This finding is explained by the anesthetic effect on the nerve branches in the groin, which lasted 24 hours in certain cases. In QL block, this effect occurs because a large volume of anesthetic solution is administered to a fascial compartment. This phenomenon has been found in countless studies on the effect of US-guided blocks, primarily TAP blocks and QL blocks, as an adjuvant to general anesthesia for laparoscopic procedures or as a single anesthetic technique in open surgery^10,34,42,43^. In 2015, Meyer *et al*. analyzed TEP inguinal hernia repair under general anesthesia with a laryngeal mask airway and TAP block to reduce the length of hospital stay; the results showed that this option was safe and reproducible^39^. The results of Aveline *et al*., who compared TAP block and ilioinguinal/iliohypogastric nerve block in open inguinal hernia repair, showed that the former afforded better postoperative pain relief and reduced the indication of opioids^44^.

In a randomized study with patients receiving TEP hernia repair under general anesthesia with or without a TAP block, Kim *et al*. found lower pain scores and reduced analgesic intake in the group that received a TAP block^40^.

Blanco *et al*. conducted a study in 2016 that compered a TAP block to a QL block as an analgesic treatment after cesarean section. The results showed that the analgesic effect associated with QL block was longer, with a consequent reduction in the administration of opioids^45^.

The fact that early postoperative pain was lower among patients who underwent QL block enabled hospital discharge 6 hours after surgery. Their pain was also lower 24 hours after discharge, which contributed to improved recovery as a function of several factors including early ambulation, the absence of paralytic ileus, and no need for stronger analgesics (e.g., opioids).

Numerous studies analyzing the cost of hernia repair are favorable to open surgery because it can be performed under locoregional anesthesia. The cost of laparoscopic surgery under general anesthesia combined with 24-hour hospital stay is higher. When adding the qualitative advantages of laparoscopic surgery, i.e., locoregional anesthesia (without orotracheal intubation) and 6-hour hospital stays, we obtained the best cost-benefit ratio among all available surgical and anesthetic techniques^11,46^.

## Conclusions

QL block is a safe and effective option for patients undergoing TEP inguinal repair given the observed reduction in early postoperative pain, briefer hospital stay, and decreased anesthesia and hospital costs.

## References

[1] Gong K (2011). Comparison of the open tension-free mesh-plug, transabdominal preperitoneal (TAPP), and totally extraperitoneal (TEP) laparoscopic techniques for primary unilateral inguinal hernia repair: a prospective randomized controlled trial. Surg. Endosc..

[2] Group TH (2018). International guidelines for groin hernia management. Hernia.

[3] Pisanu A, Podda M, Saba A, Porceddu G, Uccheddu A (2014). Meta-analysis and review of prospective randomized trials comparing laparoscopic and Lichtenstein techniques in recurrent inguinal hernia repair. Hernia.

[4] Bittner R (2011). Guidelines for laparoscopic (TAPP) and endoscopic (TEP) treatment of inguinal Hernia [International Endohernia Society (IEHS)]. Surg. Endosc..

[5] Symeonidis D (2014). Prospective non-randomized comparison of open versus laparoscopic transabdominal preperitoneal (TAPP) inguinal hernia repair under different anesthetic methods. Surg. Today.

[6] Köckerling F (2017). TEP or TAPP for recurrent inguinal hernia repair—register-based comparison of the outcome. Surg. Endosc..

[7] Neumayer L (2004). Open Mesh versus Laparoscopic Mesh Repair of Inguinal. Hernia. N. Engl. J. Med..

[8] Dulucq JL (1992). Treatment of inguinal hernia by insertion of a subperitoneal patch under pre-peritoneoscopy. Chirurgie.

[9] Langeveld HR (2010). Total Extraperitoneal Inguinal Hernia Repair Compared With Lichtenstein (the LEVEL-Trial). Ann. Surg..

[10] Dahlstrand U, Sandblom G, Ljungdahl M, Wollert S, Gunnarsson U (2013). TEP under general anesthesia is superior to Lichtenstein under local anesthesia in terms of pain 6 weeks after surgery: results from a randomized clinical trial. Surg. Endosc..

[11] Castoro, C., Bertinato, L., Baccaglini, U., Drace, C. a. & McKee, M. Policy Brief – Day Surgery: Making it Happen. *Eur. Obs. Heal. Syst. Policies* 1–32 (2007).

[12] Pereira JC, de R, Trugilho JCV, Eulálio JMR, Jamel N (2006). Avaliação do tratamento da hérnia inguinal sob anestesia local e sedação em 1560 pacientes. Rev. Col. Bras. Cir..

[13] Ford S, Evans R, Bishop J, Egeler C (2010). Radiological and clinical assessment of an ultrasound guided quadratus lumborum compartment block. Reg. Anesth. Pain Med..

[14] Baeriswyl M, Kirkham KR, Kern C, Albrecht E (2015). The Analgesic Efficacy of Ultrasound-Guided Transversus Abdominis Plane Block in Adult Patients. Anesth. Analg..

[15] Boretsky KR (2014). Regional anesthesia in pediatrics. Curr. Opin. Anaesthesiol..

[16] BLANCO R (2007). 271: Tap block under ultrasound guidance: the description of a “no pops” technique. Reg. Anesth. Pain Med..

[17] Børglum J, Gögenür I, Bendtsen TF (2016). Abdominal wall blocks in adults. Curr. Opin. Anaesthesiol..

[18] Børglum, J., *et al*. Ultrasound-Guided Transmuscular Quadratus Lumborum Blockade. *BJA Br. J. Anaesth*. **111** (2013).

[19] Ueshima H, Otake H, Lin J-A (2017). Ultrasound-Guided Quadratus Lumborum Block: An Updated Review of Anatomy and Techniques. Biomed Res. Int..

[20] Muysoms FE (2016). A prospective, multicenter, observational study on quality of life after laparoscopic inguinal hernia repair with ProGrip laparoscopic, self-fixating mesh according to the European Registry for Abdominal Wall Hernias Quality of Life Instrument. Surgery.

[21] Favaro ML (2017). Total extraperitoneal laparoscopic inguinal hernia repair making exclusive use of the square lumbar muscle anestesia guided by ultrasound. Hernia.

[22] Furtado M (2019). Systemization Laparoscopic inguinal hernia repair (TAPP) based on a new anatomical concept:inverted Y and five triangles. Arq. Bras. Cir. Dig.

[23] Meyer A, Dulucq J-L, Mahajna A (2013). Laparoscopic hernia repair: nonfixation mesh is feasibly?. Arq. Bras. Cir. Dig..

[24] Aldrete JA (1995). The post-anesthesia recovery score revisited. J. Clin. Anesth..

[25] Ferreira-Valente MA, Pais-Ribeiro JL, Jensen MP (2011). Validity of four pain intensity rating scales. Pain.

[26] Eisenberg D, Hwa K, Wren SM (2015). Telephone Follow-Up by a Midlevel Provider After Laparoscopic Inguinal Hernia Repair Instead of Face-to-Face Clinic Visit. JSLS J. Soc. Laparoendosc. Surg..

[27] Myers E, Browne KM, Kavanagh DO, Hurley M (2010). Laparoscopic (TEP) Versus Lichtenstein Inguinal Hernia Repair: A Comparison of Quality-of-Life Outcomes. World J. Surg..

[28] Bittner R (2015). Update of guidelines on laparoscopic (TAPP) and endoscopic (TEP) treatment of inguinal hernia (International Endohernia Society). Surg. Endosc..

[29] Dhankhar DS (2014). Totally extraperitoneal repair under general anesthesia versus Lichtenstein repair under local anesthesia for unilateral inguinal hernia: a prospective randomized controlled trial. Surg. Endosc..

[30] Eker HH (2012). Randomized Clinical Trial of Total Extraperitoneal Inguinal Hernioplasty vs Lichtenstein Repair. Arch. Surg..

[31] Ris F (2014). Addition of transversus abdominis plane block to patient controlled analgesia for laparoscopic high anterior resection improves analgesia, reduces opioid requirement and expedites recovery of bowel function. Ann. R. Coll. Surg. Engl..

[32] Hartford L (2018). Standardization of Outpatient Procedure Narcotics: A Prospective Non-Inferiority Study to Reduce Opioid Use in Outpatient General Surgical Procedures. J. Am. Coll. Surg..

[33] Narouze, S. N. & Guirguis, M. Ultrasound-Guided Transversus Abdominis Plane (TAP) Block. In *Atlas of Ultrasound-Guided Procedures in Interventional Pain Management* 157–160. 10.1007/978-1-4939-7754-3_15 (2018).

[34] Arora S (2016). Transversus abdominis plane block for laparoscopic inguinal hernia repair: a randomized trial. J. Clin. Anesth..

[35] Mishra M, Mishra SP (2016). Transversus abdominis plane block: The new horizon for postoperative analgesia following abdominal surgery. Egypt. J. Anaesth..

[36] Tzovaras G (2012). Long-term results after laparoscopic transabdominal preperitoneal (TAPP) inguinal hernia repair under spinal anesthesia. Hernia.

[37] Hausman MS, Jewell ES, Engoren M (2015). Regional Versus General Anesthesia in Surgical Patients with Chronic Obstructive Pulmonary Disease. Anesth. Analg..

[38] Zacharoulis D (2009). Laparoscopic transabdominal preperitoneal repair of inguinal hernia under spinal anesthesia: a pilot study. Am. J. Surg..

[39] Meyer A, Bonnet L, Bourbon M, Blanc P (2015). Totally extraperitoneal (TEP) endoscopic inguinal hernia repair with TAP (transversus abdominis plane) block as a day-case: A prospective cohort study. J. Visc. Surg..

[40] Kim MG (2012). The analgesic effect of ultrasound-guided transverse abdominis plane block after laparoscopic totally extraperitoneal hernia repair. Korean J. Anesthesiol..

[41] Muzio G, Bernard K, Polliand C, Rizk N, Champault G (2006). Impact of peritoneal tears on the outcome and late results (4 years) of endoscopic totally extra-peritoneal inguinal hernioplasty. Hernia.

[42] Planells Roig M (2011). Dolor percibido, consumo de analgésicos y recuperación de las actividades de la vida diaria en pacientes sometidos a hernioplastia inguinal ambulatoria laparoscópica tipo TEP versus hernioplastia Lichtenstein en régimen ambulatorio. Cirugía Española.

[43] Venkatraman R, Ranganathan Jothi A, Sakthivel A, Sivarajan G (2016). Efficacy of ultrasound-guided transversus abdominis plane block for postoperative analgesia in patients undergoing inguinal hernia repair. Local Reg. Anesth..

[44] Aveline C (2011). Comparison between ultrasound-guided transversus abdominis plane and conventional ilioinguinal/iliohypogastric nerve blocks for day-case open inguinal hernia repair. Br. J. Anaesth..

[45] Blanco R, Ansari T, Riad W, Shetty N (2016). Quadratus Lumborum Block Versus Transversus Abdominis Plane Block for Postoperative Pain After Cesarean Delivery. Reg. Anesth. Pain Med..

[46] Swanstrom LL (2000). Laparoscopic Hernia Repairs. Surg. Clin. North Am..

